# A New Twist to a Chronic HCV Infection: Occult Hepatitis C

**DOI:** 10.1155/2015/579147

**Published:** 2015-06-24

**Authors:** Bashar M. Attar, David Van Thiel

**Affiliations:** ^1^Division of Gastroenterology and Hepatology, Cook County Health and Hospitals System, 1901 West Harrison Street, Chicago, IL 60612, USA; ^2^Rush University Medical Center, Chicago, IL 60612, USA; ^3^Advanced Liver and Gastrointestinal Disease Center, Berwyn, IL 60402, USA

## Abstract

*Background*. The prevalence of occult hepatitis C infection (OCI) in the population of HCV-RNA negative but anti-HCV positive individuals is presently unknown. OCI may be responsible for clinically overt recurrent disease following an apparent sustained viral response (SVR) weeks to years later. *Purpose*. To review the available current literature regarding OCI, prevalence, pathogenic mechanisms, clinical characteristics, and future directions. *Data Sources*. Searching *MEDLINE*, article references, and national and international meeting abstracts for the diagnosis of OCI (1990–2014). *Data Synthesis*. The long-term followup of individuals with an OCI suggests that the infection can be transient with the loss of detectable HCV-RNA in PPBMCs after 12–18 months or alternatively exist intermittently and potentially long term. The ultimate outcome of HCV infection is decided by interplay between host immune responses, antiviral therapies, and the various well-identified viral evasion mechanisms as well as the presence of HCV infection within extrahepatic tissues. *Conclusion*. The currently widely held assumption of a HCV-cure in individuals having had “SVR” after 8–12 weeks of a course of DAA therapy as recently defined may not be entirely valid. Careful longitudinal followup utilizing highly sensitive assays and unique approaches to viral isolation are needed.

## 1. Background

The last 3 decades have been phenomenal progress in the recognition, replication mechanisms, and treatment of HCV (Box 1).

These advances have enabled the treatment of chronic hepatitis C infections to undergo dramatic changes since the inception of therapy with interferon *α* 3 times/week for 48 weeks in 1991-1992 [1, 2]. Not only have the rates of a sustained viral response (SVR) increased to values approximately 90%, but the duration of therapy has been reduced from 48 weeks to 6–12 weeks with the currently utilized protocols consisting of orally administered direct acting antiviral (DAA) agents [3–5]. Most relapses following historical treatment protocols as well as current protocols occur within 1–4 weeks after the end of treatment (EOT) time point. Yet, a minority of relapses occur months to years later [6–8]. Although the origin of these late relapses is uncertain, an increasing amount of data suggests that they may represent activation of an occult hepatitis C virus infection (OCI) [9–13]. OCI was first reported by Pham et al. [9] in anti-HCV positive patients recovered after a self-limited (untreated) episode of hepatitis C and in individuals with SVR due to interferon (IFN) treatment who had normal liver enzyme [9]. In the same year, Castillo et al. [10] described HCV-RNA presence in anti-HCV negative patients with elevated liver enzymes. Therefore, these authors discovered OCI and recognized the two different forms of this occult infection [10] (Table 1). The prevalence of OCI in the population of HCV-RNA negative but anti-HCV positive individuals is presently unknown but has stimulated considerable interest [12, 13]. Currently, the detection of HCV-RNA in liver tissue or in peripheral blood mononuclear cells (PBMCs) occurring in an individual with undetectable HCV-RNA in serum represents the gold-standard for the identification of an occult HCV infection. The definition of an OCI has been expanded by the identification of HCV-RNA in extra hepatic tissues of anti-HCV negative individuals [9–13]. Currently, OCI is characterized by undetectable levels of HCV-RNA in serum/plasma utilizing current laboratory assays with the identification of HCV-RNA in PBMCs and/or liver tissue utilizing molecular assays and analyte treatment protocols resulting in enhanced sensitivity [9–15]. A major limitation of identifying OCI cases is the lack of standardized universal sensitive method for detecting HCV-RNA in these studies. Thus, the detection rates of HCV-RNA in PBMCs have varied from 0% to 50% [16–19]. Using a less sensitive assay to detect HCV-RNA, Carreño et al. [16] found that 6 of 12 individuals have detectable genomic HCV-RNA (50%) in PBMCs. These authors used a quantitative, strand-specific, real-time reverse transcription polymerase chain reaction (RT-PCR) to detect genomic and antigenomic strands. The total RNA concentration from PBMCs and hepatic tissue was determined by utilizing spectrophotometry [16]. In contrast, Bernardin et al. [17] used a highly sensitive transcription-mediated amplification (TMA) test performed in duplicate assays. None of their 69 aviremic individuals with hepatitis C infection had a detectable PBMC-associated HCV-RNA. The HCV-RNA detection rates in liver biopsy specimens varied widely among studies ranging from 0% to 83% [20–23]. Haydon et al. other studies detected HCV-RNA in liver biopsy tissues in 10 of 12 individuals (83%) using RT-PCR [20]. Barrett et al. showed that none of the 33 serum PCR-negative women for HCV had a HCV in their hepatic tissue specimens using a sensitive nested PCR with an internal control [21]. Using RT-PCR, Sugiyasu et al. [22] differentiated HCV infected individuals who did not develop chronic hepatitis C (CHC) and cleared the HCV virus spontaneously and their hepatic tissue specimens were negative for HCV-RNA from those who developed CHC. Of those who did not develop CHC, 6 of 6 individuals who cleared the HCV-RNA spontaneously were found to have negative PCR testing of HCV in their hepatic tissue by using specific RT-seminested PCR which was described by Tomimatsu et al. [23]. In contrast, of the two individuals who developed CHC in their report and experienced a SVR with interferon-based therapy, one had persistent HCV genome in the liver [22].

There are only a few studies which applied high stringency methods for HCV-RNA detection and calibrated their assays as well as utilization of sequence analysis or nucleic acid hybridization probe amplification [24–34]. Pham et al. [27] reported the original finding of occult hepatitis C infection (OCI) subsequently used these highly sensitive assays such as the hybridization to support their findings with assays capable of detecting HCV-RNA to ≤10 vge/mL (≤3 IU/mL) or ≤ 5 vge/ug (≤1.5 IU/ug) [26, 28]. Despite what is believed to be a cure and a complete eradication of HCV-RNA either spontaneously or after therapy, low levels of HCV-RNA persisted in plasma (100–200) virus genome equivalent (vge) or genome copies/mL, or up to 100 vge/ug total viral RNA in both PBMCs and hepatic tissue specimens [27–30]. Furthermore, it was shown that individuals with OCI who have CHC have both a unique cytokine pattern as well as a gene expression pattern which differs from those who do not manifest an OCI [24, 31].

Others have utilized ex vivo stimulation of PBMCs with mitogens to identify the cause of OCI defined as the presence of HCV-RNA positive and negative strands and HCV proteins [9, 25, 27, 35]. To further improve the detection of OCI, screen serial samples collected from the same individuals at different periods [25, 27, 31]. The quality and the amount HCV-RNA recovered of HCV-RNA is crucial to the identification of the viral RNA and require specific handling of the specimens [36].

Importantly, the detection of low levels HCV-RNA in serum following an apparent “SVR” has been implicated in HCV infectivity by MacParland et al. [37] who reported exposure of human normal T cells to trace HCV levels found in plasma and supernatants from cultured PBMCs obtained from these individuals with apparent SVR post-Peg-IFN/RBV therapy for periods as long as 72 months could establish a* de novo* HCV infection with replication of HCV virions in vitro. Only 20–50 copies of HCV were sufficient to produce infection of these human T cells [37]. Similarly, Katayama et al. have shown that 20 copies of HCV-RNA prepared by dilution of serum collected during the pre-acute phase of hepatitis C of an infected chimpanzee can result in an infection in inoculated chimpanzees without an increase in ALT levels [38].

Despite studies supporting the evidence of presence of OCI, there continues to be a controversy by some authors who challenged the existence of OCI [17, 39–43].

## 2. Clinical Characteristics of OCI

The use of highly sensitive assays and procedures that enrich HCV in test samples by amplifying the viral RNA present in the analyte material obtained from plasma, PBMCs, liver tissue, or other tissue sites such as the thyroid, salivary glands, pancreas, kidney, and even the brain, where HCV-RNA has been shown to exist, defines OCI [12–15, 44, 45]. Importantly, HCV-RNA replication manifested by the presence of the negative strand of HCV-RNA has been reported in some of these extrahepatic sites [44, 46] including PBMCs [9]. The implications of these findings are clinically and critically important. Thus, the use of the terms “cure” and “total eradication” of HCV following treatment occurring in association with a currently defined SVR may not be appropriate at least in a minority of cases.

The observation that HCV can infect extra-hepatic tissues in addition to the liver particularly PBMCs and other tissues which represent privileged reservoirs for HCV that are potentially responsible for the development of an OCI has reinvigorated the interest in HCV replication. The continued replication of genomic HCV in these extrahepatic tissue sites at a rate well below that which occurs in the liver during active infection at a time when HCV-RNA is undetectable in the serum most probably is the origin of OCI [16, 32, 47–52].

There are currently two immune-viral/clinical distinct forms of OCI both of which were originally reported by Pham et al. [9] and Castillo et al. [10]:

1. Individuals with detectable HCV-RNA and anti-HCV, in the absence of elevated liver enzymes, which occurs in otherwise successful antiviral therapy or a self-limiting episode of hepatitis C. These individuals have HCV-RNA detected in their serum, PBMCs, and liver. And have been labeled as having “secondary OCI” indicating the presence of residual HCV infection persisting despite spontaneous resolution of hepatitis C or achieving apparent “SVR” following HCV therapy [9].
2. Individuals presenting with HCV-RNA positivity but anti-HCV negative with elevated liver enzymes [10].

The diagnosis of these OCI in anti-HCV positive individuals with normal liver enzymes was originally based on investigations of PBMC and plasma but not liver biopsies. Subsequently, hepatic tissues were investigated in these cases by Pham et al. 6–66 months after HCV therapy [9, 50] and by Radkowski et al. 41–98 months following HCV treatment [28].

## 3. HCV OCI Experience

Although it is not particularly surprising to detect HCV using highly sensitive amplifying assays for HCV-RNA, Chen at al. reported the detection of HCV and continued viral replication during ongoing antiviral treatment of patients with PegIFN/R at a time when viral RNA was undetectable in the serum. These data help to explain the late reappearance of detectable HCV-RNA by currently available clinical tests in individuals having had an apparent SVR in as many as 11.6% of the studied cases [46].

Specifically, a total of 122 patients, suspected of having an occult HCV infection following a putative SVR, were studied utilizing the detection of anti HCV core and methods to detect HCV-RNA either in the liver or PBMC [53]. Anti-HCV core protein was detected in the serum and PBMCs in 36% of the cases. After ultracentrifugation of the serum, 70/122 (57%) were reported to have identifiable HCV-RNA in their serum and 74/122 (61%) had HCV-RNA identifiable in their PBMCs. When these two approaches for the detection of OCI were combined, 91% of the patients (111/122) were positive in at least one of the two assays. In light of these results, occult HCV infection was reported to be present in as many as 91% of patients, who are anti-HCV positive and have had a SVR defined as having no HCV-RNA detected in their serum utilizing currently available commercial assay methods [53].

These data strongly suggest that occult HCV infection may be present in at least some individuals who either spontaneously or after antiviral treatment have “cleared” virus from their serum [54–56].

Ansaldi et al. [57] reanalyzed the data of Castillo et al., [14] who reported a prevalence of 2.7% anti-HCV positive individuals among the Italian population and determined that the likelihood of a false negative result for anti-HCV was essentially zero. In a study reported by de Marco et al., [11] the prevalence of OCI infection in “healthy” Italian population was 3.3% of individuals who were HCV-Ab negative and serum HCV-RNA negative and had detectable HCV-RNA in their PBMCs. Although the number of participants in this study was relatively small with only 3.3% being positive for HCV-RNA among a total of 276 subjects, it supports the existence of OCI not only in anti-HCV positive individuals but also those who are anti-HCV negative. Moreover, this finding suggests that HCV infection might be spread from an apparent “healthy” individual with an OCI to an HCV naive individual who may subsequently develop clinical disease [45]. These latter observations are consistent with the observation that despite multiple approaches to reduce the risk of leukocyte-transfusion-related HCV infection, to include leukocyte depletion, the efficacy of such procedures at reducing the already low risk of virus transmission with blood product transfusion continues to occur albeit rarely [45, 57, 58].

De Marco et al. [59] evaluated the prevalence of OCI in a larger cohort (314) of infectious liver disease-free (ILDF) subjects having no clinical evidence for HBV, HCV, CMV, EBV, and other viral illness, who were followed to determine the natural history of OCI. The prevalence of OCI in the ILDF subjects studied was 1.27%, while an even higher prevalence of OCI (28%) was identified in HBV carriers. No specific HCV genotype has predominated in the subjects with OCI studied to date [59].

The long-term followup of individuals with an OCI suggests that the infection can be transient with the loss of detectable HCV-RNA in PPBMCs after 12–18 months or alternatively exist intermittently and potentially long term [59]. Confirmation of these studies with a longer follow-up period is needed to define the clinical characteristics and prevalence of OCI, the rate of OCI resolution, and/or the rate of reactivation either as intermittent OCI or overt clinical infection. These data of Barril et al. [60] of intermittent OCI are consistent with the data of Castillo et al., [61] who reported a series of 37 cases having an OCI manifested by abnormal liver enzymes for a minimum of 12 months with the persistence of very low levels of HCV-RNA intermittently in PBMCs and serum in the subjects they followed for a mean of 55.7 months.

Studies with 10 years of followup after achieving an apparent SVR have reported the presence of small amounts of HCV-RNA in individuals' serum or plasma, PBMCs, and/or hepatic tissue associated with normal liver enzymes indicating the presence of OCI by several authors [9, 28–30, 62, 63] with a range of 10–100% depending mostly on the sensitivity of the methods utilized to detect HCV-RNA [19]. Despite significant improvement in liver histology after achieving a SVR, minimal to mild disease activity associated with focal hepatic necrosis, lymphocytic infiltration, and various stages of fibrosis have been reported [28, 30, 50].

Taken together, these studies suggest that the currently widely held assumption of a HCV-cure in individuals having had “SVR” after 8–12 weeks of a course of DAA therapy as recently defined may not be entirely valid. Careful longitudinal followup utilizing highly sensitive assays and unique approaches to viral isolation are needed to define the frequency of both OCI and a true SVR in individuals receiving these shorter courses of therapy.

## 4. OCI in Immune Impaired Individuals

In similar but not identical studies, Barril and colleagues [60] reported prevalence of an OCI in 45% of 109 hemodialysis patients. These investigators also reported on the potential for transmission of HCV by individuals with OCI. Specifically, they found that the presence of OCI among the relatives of individuals they studied was comparable to that found among family members of patients with overt HCV infection. The Barril et al. report [60] and others [64, 65] however stand in contrast to that of other authors [39, 66] who were not able to identify cases of OCI in different cohorts of immune-suppressed subjects consisting of 28 oncohematological patients [67] and 26 kidney-transplant recipients [68].

**Figure 1:**
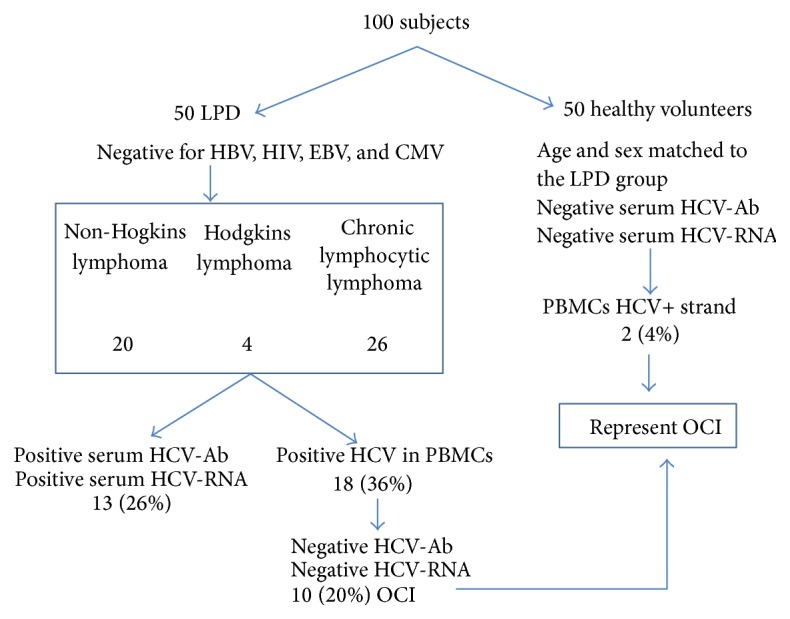
Prevalence of OCI in patients with chronic lymphoproliferative disorders (LPD). OCI was detected in 20% of the LPD group versus 4% in controls, adapted from [103].

An intriguing additional question is what is the relationship between the existence of an OCI and the subsequent development of either a lymphoproliferative disease [69] or cryoglobulinemia [70, 71]? Youssef et al. [72] investigated the occurrence of OCI in Egyptian patients with variety of lymphoproliferative disorders (LPDs) (Figure 1). Their study included 100 subjects consisting of 50 consecutive cases with a newly diagnosed lymphoproliferative disease and 50 healthy anti-HCV negative controls. 13 of the 50 (26%) with an LPD group were positive for anti-HCV and all were HCV-RNA positive as well. HCV-RNA was detected in PBMC in 18 of the 50 LPD patients (36%). Importantly, ten of these 18 cases were negative for both anti-HCV and HCV-RNA in their serum and therefore represented true cases of OCI. In the healthy anti-HCV negative controls, the HCV-positive (genomic) strand was found in PBMC in 2 (4%) of those studies thereby fulfilling the criteria for an OCI. The HCV genotype in all 12 cases of OCI was genotype 4, the predominant genotype present in Egypt [72].

Farahani et al. [73] identified OCI in 2 (1.9%) of 104 Iranian patients with LPD. The authors suggested testing for genomic HCV-RNA in PBMCs in the absence of liver biopsy in individuals with LPD [73].

Similarly, genomic and antigenomic HCV-RNA was detected in the PBMCs of 5 of 9 (56%) individuals who had chronic hepatitis C with mixed cryoglobulinemia and achieved SVR after receiving antiviral therapy [71].

**Figure 2:**
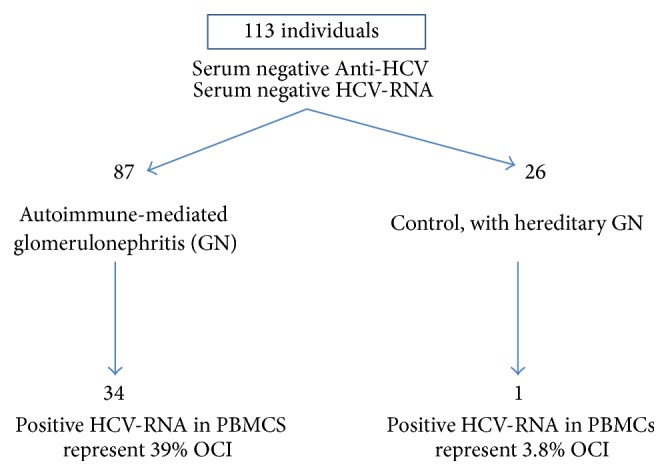
The prevalence of OCI in patients with primary and secondary glomerular nephropathies. There is a significant increased risk (odd ratio 13.29) of OCI in patients with immune mediated glomerulonephritis versus the controls [74].

Castillo et al. [74] reported that occult HCV-RNA (detectable viral RNA in peripheral blood mononuclear cells or in serum after ultracentrifugation) was found in 34 of 87 patients with immune mediated glomerulonephritis versus 1 of 26 control patients with hereditary glomerulonephritis (Figure 2). Importantly, all of the cases studied were HCV-RNA negative in serum utilizing a commercially available assay [75]. A multivariate analysis demonstrated a significantly increased risk of occult HCV in patients with immune mediated glomerulonephritis versus the controls with heritable forms of glomerulonephritis (odds ratio of 13.29). Furthermore, the rate of progression to end-stage renal disease tended to be faster in the OCI group of patients as compared to those without an OCI [74].

Importantly, among patients with LPD, Kisiel et al. [76] studied 77 anti-HCV negative patients in Poland. HCV-RNA was detected by RT-PCR in 13% of the plasma samples, in 16.9% of the PBMCs samples, and in the bone marrow of 28.6% of the cases [76].

## 5. Potential Pathogenic Mechanisms Resulting in an OCI (Box 2)

Pham et al. [25] identified the subsets of immune cells involved in the HCV infection in individuals with CHC and others with OCI. They isolated immune cell subtypes from 7 patients with CHC and 7 individuals with occult infection and analyzed them for HCV-RNA. While HCV-RNA occurred at a similar frequency in all of the various cell subtypes of individuals with CHC, monocytes had the greatest viral load. In contrast, B cells in individuals with OCI manifested a tendency toward having higher HCV quantities compared to monocytes. The detection of HCV nonstructural protein 5A and HCV variants that were not found in plasma defined HCV replication in different immune cell types which presumably serve as reservoirs of the virus in individuals with OCI [25].

Chen et al. [46] have reported that mononuclear cells, including T lymphocytes, are targets for HCV and these cells are reservoirs of replicating HCV regardless of the presence or absence of either symptomatic or asymptomatic infection. As these cells are readily accessible, they can be utilized to evaluate the status of extra hepatic HCV replication during an active infection during the course of antiviral treatment and after the apparent development of a putative SVR. In this context, the detection of HCV positive cells and in some cases the replicative negative viral strand in PBMCs confirms the role of these cells as a HCV reservoir both during ongoing antiviral treatment and most importantly after its completion and thereby enabling the identification of cases of OCI [46].

The ultimate outcome of HCV infection is decided by interplay between host immune responses, antiviral therapies, and the various well-identified viral evasion mechanisms as well as the presence of HCV infection within extrahepatic tissues (reservoirs of occult infection) [77–81].

Quiroga et al. [77] showed that a strong and sustained HCV-specific CD4+ and CD8+ T cell responses are essential for the resolution of HCV infection. Importantly, the authors [77] have considered these specific T cell responses as immune markers for predicting prior HCV exposure, recovery, or OCI. Depletion of CD4+ T cells plays a major role in the persistence of HCV infection while depletion of CD8+ T cells was associated with delayed clearance of HCV-RNA [82, 83]. Specifically, CD8+ T cell responses recognize viral antigens in the context of HLA class I, primed during acute HCV infection and considered to be critical for subsequent viral clearance [84]. In individuals, who have high levels of viral replication, the lack of viral control can be attributed to the development of viral mutations which potentially result in an enhanced viral infection of extra hepatic tissues that have a reduced rate of viral replication, cell surface expression of viral antigens, and subsequent recognition and elimination by CD8+ antiviral responses. Such mutational escape mechanisms have been shown to occur in a predictable fashion in the context of specific HLA profiles and are highly likely to play an important role in the persistence of HCV infection to include cases of OCI [80, 84–86].

The cytokine balance between Th1/Th2 may also be a factor in the development of OCI cases [77, 84–89]. Mousa et al. [87] investigated the role of cytokine profiles including cytokines related to T-cell immunoregulation in the development of OCI. They compared the cytokines responses between OCI and those with an identifiable chronic hepatitis C infection (CHC). They found that Th1 cytokines are significantly greater in cases of CHC patients than in those with OCI or control noninfected individuals. In contrast, individuals with an OCI had higher serum interleukin- (IL-) 4 and levels than did those with CHC and the healthy controls. Serum levels of IL-10 were higher in both OCI and CHC groups compared with control. Thus, the presence of a cytokine shift towards enhanced Th2 expression in OCI cases may have resulted in decreased serum level of IFN-*γ* and IL-2 cytokines associated with subsequently immune clearance of the viral infection [77, 84, 86–88]. Importantly, the maintenance of polyfunctional HCV-specific Th1 T cells, CD4+, and CD8+ memory T cells resulted in spontaneous clearance of HCV and better outcome of treatment of the HCV infection [88]. Pham et al. [24] reported that PBMCs from OCI individuals have higher levels of IFN-*α*, IFN-*γ*, and TNF-*α* but less IL-10 than CHC patients. Stronger HCV replication in immune cells was noted in individuals with lower transcription of IFN-*α*, IFN-*γ*, and TNF-*α*, but higher levels of IL-10. These data support the presence of distinct cytokine pattern between OCI and CHC.

Plasma interferon-gamma-inducible protein-10 (IP-10) levels have been shown to be associated with early but not sustained virological response during treatment of acute and chronic HCV infection. Baseline IP-10 has been associated with viral levels but not treatment outcome. Individuals with high baseline IP-10 greater than 600 pg/mL rarely clear virus [90].

Several investigators have shown that HCV-infected subjects can harbor HCV quasispecies in their PBMCs that are not detectable in plasma. In such cases, it is presumed that the virus quasispecies identified in plasma is more efficiently replicative and as a result has created less space for the less actively replicating virus in plasma such that they become sequestered in nonhepatic reservoirs for virus [28, 32, 46–49]. A potential additional explanation for these differences in the distribution of viral quasispecies may be related to the presence of viral mutations that confer a unique cellular tropism for PBMCs [91–95]. Specifically, studies of the hyper-variable E2 region of HCV have shown that viruses with specific mutations in this region are found more frequently in B lymphocytes and monocytes than in the serum [46, 79]. This suggests that the identification of HCV species with E2 polymorphisms is likely enriched in PBMC as compared to plasma and liver tissue as may represent a unique pathogenic basis for the development of a subsequent OCI. These viral variants can be found before and during antiviral therapy and may persist well in these extrahepatic sites long after a presumed SVR has been achieved.

The IL28B gene locus encodes for IFN-*κ*3, a member of type III IFN family [44, 96–98]. Type III IFN family shares downstream signaling pathways with type I IFNs. Both interferons initiate Jak/signal transduction and subsequent activation of transcription (STAT), and the subsequent intracellular pathways that upregulate the transcription of IFN-stimulated genes (ISGs), resulting in an enhanced antiviral response. Recently, several large genome-wide association studies have identified several single nucleotide polymorphisms (SNPs) linked to interferon lambda 3 (IFN*λ*3) that are associated with either a high likelihood of viral clearance with IFN treatment or the spontaneous resolution of HCV infection. These observations generated intense research activity and strongly supported the identification of IFN*λ*3 genetic variants which serve as important predictive biomarkers of IFN treatment of HCV [99–101].

Specifically these studies have demonstrated that the presence of a single nucleotide polymorphism (SNP) at the IL28B locus, specifically the presence of the T alleles of rs12979860 or the G alleles of rs8099917 is associated with a reduced response to PegIFN/RBV therapy and importantly also an increased prevalence of PBMC infection (OR: 3.564; 95% CI: 1.114–11.40, *P* = 0.0437) [98, 102–104].

The use of IFN*λ* family that induces a cellular antiviral and immunomodulatory response that is specific for the liver but has little or no activity in extrahepatic tissues as opposed to the standard IFN-*α* may more contribute a subsequent greater rate of OCI [104].

Another potential explanation for the failure to clear HCV-RNA from PBMC is a host-based resistance to the therapeutic actions of ribavirin (RBV). Ibarra et al. [105] have shown that cellular uptake of RBV into PBMCs decreases over time and may explain at least in part why PBMCs become a reservoir of HCV and potentially contributes to the development of treatment failure, disease recurrence, and in some cases the development of an OCI. In addition, Bartolomé et al. [49] have shown that HCV-infected B cells exhibit a IFN-resistant phenotype. These findings strongly suggest that the degree to which a viral infection of PBMC occurs contributes to the selection of distinct viral variants that persist in these cells and subsequently serve as the source of both late disease recurrences and OCI.

**Figure 3:**
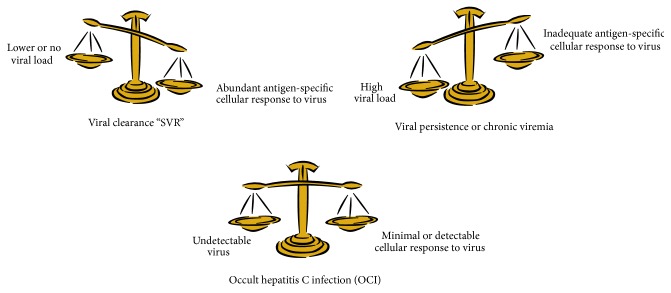
Balanced immunologic response to virus.

It has been recognized only recently that a balance exists between mobile antigens (viruses) and the antigen-specific T cells that govern the immunologic response or nonresponse to an infection agent. Specifically, Zinkernagel study experimental viral disease model noted that immunity to the virus as well as other intracellular infectious organisms is produced and maintained by a staged migration of the antigen (virus) and activated T cells and the outcome of this migration is governed by the cyo tokine, growth factors, and other interacting components that modulate viral and T cell behavior and the localization of specific features of lymphoid tissues. After an initial viral response, residual virus can become protected in nonlymphoid locations that are inaccessible to humoral and cellular effector mechanisms. Viruses in these locations are unrecognized until they migrate back to lymphoid tissues insufficient numbers to result in disease elimination (SVR) or disease reactivation (relapse) [106–109] Figure 3.

## 6. Clinical Relevance

Several small studies have described patients who have achieved an apparent SVR but continue to have HCV-RNA detected in their peripheral blood mononuclear cells, lymphocytes, and macrophages utilizing more sensitive research assays [9–13, 110–114]. More importantly, the negative strand of HCV-RNA, consistent with viral replication, has been identified in the liver and peripheral blood mononuclear cells of patients having had an apparent SVR [115–117]. It needs to be pointed out that these highly sensitive reverse transcription polymerase chain reaction assays used in these investigational studies are not utilized clinically. Whether the virus detected with these techniques actually identifies replication-competent virus and therefore virus having clinical significance is not entirely clear.

Rahman et al. [118] have reported that a SVR to current IFN treatment regimens is durable in most patients, but not all and that late relapses can occur during long-term followup. Importantly, they reported that this finding occurs more often in patients with cirrhosis.

Similarly, Sood et al. [119] reported 100 patients, who achieved a SVR and were followed up for durations ranging from 6 months to 8 years. 8 of these 100 patients developed a late recurrence. The late relapses were more frequent in patients with cirrhosis (5/28 [18%] versus 3/72 [4%] without cirrhosis; *P* = 0.037). The data demonstrate that while a SVR is durable in most patients, some individuals particularly those with cirrhosis experience late relapses. Whether these late relapses are a result of OCI and whether the frequency of OCI in cirrhotics as suggested earlier is greater than in noncirrhotics and accounts for cases of OCI as well as cases of late relapses remain to be determined [119].

More recently, Rutter et al. [120] investigated the durability of a SVR in patients treated with peginterferon-*α*2a/ribavirin in combination with a direct acting antiviral agent. One hundred and three patients with chronic hepatitis C genotype 1 infection [f/m: 34/69; GT-1b: 67 GT-1a: 34, GT-4: 2; mean age: 47.6 years (45.5–49.7; 95% CI)] who achieved a SVR to triple therapy were followed. Two cases of a late relapse (2/103, 1.9%; 95% CI: 0.24–6.8) were observed. One of these two patients was cirrhotic. The relapses occurred 8 and 12 months after cessation of their antiviral therapy. Subsequent cloning sequence studies identified the genomic sequence in both patients as being identical to that of their original virus. In addition, resistance analysis revealed the absence of identifiable viral resistance to the therapeutic agents utilized in these two patients. These data support the conclusion that the relapses represented cases of clinically reactivated OCI rather than being a result of a* de novo* infection [120].

Gordon et al. [121] evaluated the long-term HCV-RNA outcomes following a defined SVR in 121 hemodialysis patients reported in 20 studies. The probability of remaining HCV-RNA negative was 86% (95% confidence interval 77% to 96%) for patients followed for 48 months after their putative SVR. Fourteen relapsed, considering the data from Rutter et al., [120] it would appear that at least some of these 14 cases represented reactivation of an OCI in an immunosuppressed population.

Giannini et al. [122] evaluated a cohort of 231 chronic HCV patients who had at least 48 weeks of followup after achieving a SVR to PEG-IFN and ribavirin. The median duration of followup after the SVR time point was 164 weeks (3.2 years) and exceeded 5 years in 30% of the study population. All 231 patients underwent clinical, biochemical, and virological evaluations every 6 months during their followup. The original sustained virological response was maintained in 211 patients (91%). HCV-RNA became positive in two patients (<1%) within 1 year after the SVR, and in an additional 18 patients (8%) HCV-RNA was transiently identified in the serum on at least one follow-up evaluation using a commercial assay with an HCV-RNA detection limit of 50 IU/mL. These investigators reported that the clinical outcome of the 231 patients studied did not differ significantly between those with persistently negative and those who were transiently positive for HCV-RNA in serum following an earlier SVR. Nonetheless, as a result of their finding, these investigators recommended biannual HCV-RNA determinations in all patients after a putative SVR to identify cases with a late virological relapse presumably occurring as a result of an OCI [123].

It has been suggested that clearance of identifiable HCV from liver tissue and/or PBMC utilizing highly sensitive reverse transcription and nested polymerase chain reaction assays may be a better indicator of a long-term sustained response than the absence of HCV-RNA in serum or plasma [56, 115–117, 124, 125]. Although the relevance of HCV-RNA detection in PBMCs alone, or in the liver in the absence of serum positivity, as well as other tissues is currently poorly understood, the ability of HCV to replicate in these extra hepatic cells, raises questions about the potential transmission risk to the liver from these sites and to other individuals as a result of blood exposure [123, 126–129]. Whether the incidence of OCI will increase with the recent shorter durations of treatment with DAA (6–24 weeks) and the revised shorter definition of a SVR of 8–12 weeks remains to be determined but is a concern. Relevant to this question, Dhaliwal and Nampoothiri [130] queried whether DAAs, which inhibit HCV by interrupting its life cycle without enhancing the individual's immune response, suppresses viral replication in the liver but not in extrahepatic sites which have reduced rates of viral replications as manifested by very much reduced viral load determinations and thereby are less susceptible to treatment with DAA [130]. Ultimately, long-term studies to confirm the durability of a SVR after the use of interferon-free DAA treatment regimens need to be determined.

## 7. Prognosis of OCI

Berasain et al. [131] reviewed 1075 consecutive liver biopsies from anti-HCV, HBsAg-negative patients with unexplained ALT elevations (defined as >1.5 upper limit of normal on two separate occasions). 27% of the cases were positive for either HBV-DNA or HCV-RNA utilizing highly sensitive techniques. The presence of a viral genome in the circulation, be it either HBV DNA or HCV-RNA, predicted clinical disease. Interestingly, the individuals with “cryptogenic” liver disease, who tested positive for either virus using the more sensitive research techniques, had a higher prevalence of cirrhosis than did those who tested negative for viruses [131].

In 100 patients with abnormal liver injury tests of unknown etiology, Castillo et al. [45] found detectable HCV-RNA in the liver tissue of 57 cases which they identified as cases of OCI. Of these 57 patients, 20 (35%) had histologic evidence of hepatic necroinflammation that occurred at a frequency significantly greater than that found in the 37 patients without intrahepatic HCV-RNA (14%). In addition, the prevalence of cases with clinically significant fibrosis (F3 or F4) was significantly greater in those identified as having an OCI (17.5%) than in those without an OCI (2.3%) [45].

## 8. The Future

Yet to be fully established is whether current and more importantly the therapies that are in development are truly capable of eradicating HCV. The current trend to define a sustained viral response at 8, 12, or 24 weeks as opposed to 48 weeks may result in an increase in late recurrences of HCV as a result of incomplete eradication of HCV in extrahepatic tissues that serve as reservoirs of HCV and have reduced rates of viral replication and lower levels of virus and are the putative sites responsible for the development of OCI cases.

As mentioned above, Angulo et al. [98] reported that genomic HCV-RNA may be present in extrahepatic sites including peripheral blood mononuclear cells (PBMCs) in 79% of patients. They also have reported that the presence of HCV-RNA in PBMC is not linked with either the serum viral load, a particular HCV genotype, the age of the patient, the duration of infection, or presence or absence of cirrhosis. However, the presence of PBMC-associated HCV-RNA was been shown to be a strong predictor of a reduced likelihood of a SVR (OR 19.4) and was associated also with a reduced rate of rapid viral response (RVR) of 60% as compared to that occurring in individuals without detectable HCV-RNA in their PBMC.

Manns et al. [132] have asked why some HCV patients, who achieve an EOT response, relapse within the 24 weeks period after discontinuation of active therapy (the time period at which a SVR is defined) or more importantly even later. They reasoned that these late relapses, occurring more than 24 weeks after an EOT response might be explained by the use of less sensitive assays for HCV-RNA resulting in a false positive apparent SVR. They evaluated the data from several different centers in Europe and the USA utilizing various assays to quantify HCV-RNA in patients treated with either IFN-a-2-b or peg-IFN a-2b. They identified 6 definite late relapsers among 636 patients with a presumed prior SVR following treatment with IFN *α*-2b and 3 definite late relapsers among 366 patients treated with PEG-IFN *α*-2b, during a 5-year followup (0.9%). A definite late relapse was defined as the finding of multiple sequential HCV-RNA tests that were positive. They also reported that there were 18 patients who received IFN *α*-2b and had nondefinite relapse, and 15 patients who were treated with PEG-IFN *α*-2b had a nondefinite relapse totaling 33 of 1002 cases (3.3%).

These individuals had a single test in which HCV-RNA was detectable followed by subsequent tests in which HCV-RNA was undetectable (possible intermittent OCI) [132].

Recently, more sensitive assays have been developed with significantly lower detection limits such as 15 IU/mL with the Roche COBAS TaqMan HCV test (Roche Diagnostics, North America, Indianapolis, IN) [133], 12 IU/mL with the Abbott Real Time HCV assay (Abbott, Chicago, IL) [134], and 10 IU/mL with the Bayer VERSANT Transcription-Mediated Amplification assay (Bayer Diagnostics, Berkley, CA) [135]. In addition, in situ hybridization [19, 136] is a useful tool for HCV detection in hepatic tissue while HCV core antigen testing [31] can be used in monitoring the HCV infection. Whether the use of these more sensitive assays would have affected the results reported by Manns et al. [132] is unknown.

Finally, current and future pharmacogenomic treatment approaches for HCV may be feasible with the incorporation of identifiable host (IFN*λ*3) and viral E2 and NS3A polymorphisms genetic variants into predictive treatment algorithms along with the currently utilized factors identified as affecting viral clearance and outcome of antiviral therapies.

## 9. Conclusions and Personal Perspective

In summary, the following conclusions can be made:

1. The evidence for the existence of the entity occult hepatitis C is substantial and growing.
2. OCI is recognized by the presence of HCV-RNA in either circulating blood mononuclear cells, the liver, or concentrated plasma triglyceride rich factions in the absence of detectable HCV-RNA in plasma.
3. The longer the duration of followup after end of treatment (EOT) is, the greater the likelihood of identifying a case of occult HCV in previously treated individuals.
4. Evidence is accumulating to suggest that cases of occult HCV occur in individuals with cryptogenic chronic liver disease consisting of both cirrhotic and noncirrhotic individuals.
5. Individuals with immune mediated disorders such as membranous glomerulonephritis, cryoglobulinemia, and non-Hodgkin's lymphoma are increasingly being identified as having an occult HCV infection.
6. Although the entity of OCI is becoming well recognized, its natural history is not yet fully defined.
7. What is known, however, is that cirrhotic individuals with immune mediated disorders are more likely to harbor an occult HCV infection and that the pace of the liver disease in such individuals appears to be accelerated as compared to those without an OCI.
8. Unique host and viral characteristics appear to contribute to the likelihood of an OCI.

These conclusions suggest the following speculations:

- Occult cases of HCV occur as a consequence of infection in unique nonhepatic immunologically protected sites or as a consequence of an infection with a HCV variant that is uniquely capable of infecting nonhepatic tissues.
- With the shorter duration of therapy with the new DAA and in the absence of interferon that has the potential to induce an immunologic attack directed at the putative “protected reservoirs” of HCV, concern for an increased prevalence of OCI in the future as a result of treatment with DAA represents a worrisome possibility.

## Figures and Tables

**Box 1:**
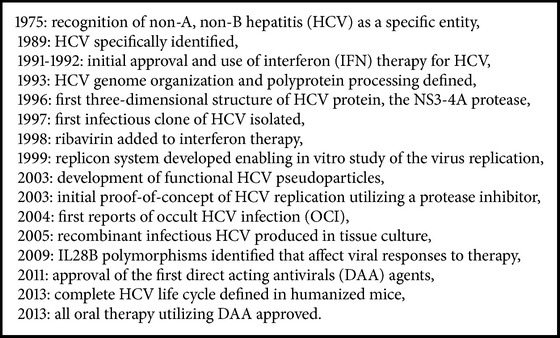
Major developments in HCV identification, replication mechanisms, life cycle sequence, and treatment.

**Box 2:**
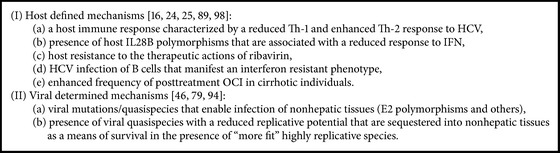
Potential pathogenic pathways to an OCI.

**Table 1:** Characteristics of occult hepatitis C infection (OCI)^+^.

	Secondary OCI^**∗**^	Cryptogenic OCI
Evidence of liver injury		
ALT elevated	No	Yes
Histological lesions		
Inflammatory: minimal to mod.	Occasional	Frequent
Fibrotic: minimal to severe	Frequent	Frequent
Organs involved		
Liver	Yes	Yes
Lymphatic system (PBMC)	Yes	Yes
Anti-HCV detection	Positive	Negative^**∗∗**^
HCV-RNA detection		
Serum	Yes	Yes
PBMC	Yes	Yes
Liver	Yes	Yes
Longevity of persistence	At least 9 years	Unknown

^**∗**^Secondary OCI: residual infection continuing after spontaneous resolution or after achieving apparent SVR following HCV therapy.

^**∗∗**^Antibodies against HCV can be detected in up to 40% in cryptogenic OCI.

^+^Adopted from Pham et al. [19], Liver International, 2010; 502-11.
